# Efficacy and tolerance of enteral nutrition in children with biliary atresia awaiting liver transplantation

**DOI:** 10.3389/fped.2022.983717

**Published:** 2022-09-02

**Authors:** Elodie Privat, Madeleine Aumar, Delphine Ley, Léa Chantal Tran, Stéphanie Coopman, Dominique Guimber, Dominique Turck, Frédéric Gottrand

**Affiliations:** ^1^Univ. Lille, Division of Gastroenterology, Hepatology and Nutrition, Department of Paediatrics, Jeanne de Flandre Children’s Hospital, CHU Lille, Lille, France; ^2^Univ. Lille, Inserm, CHU Lille, U1286 – INFINITE – Institute for Translational Research in Inflammation, Lille, France

**Keywords:** biliary atresia, enteral nutrition, nasogastric tube, stunted growth, malnutrition

## Abstract

**Objectives:**

Malnutrition is common in children with biliary atresia (BA) awaiting liver transplantation (LT). Few studies have evaluated the effectiveness of enteral nutrition (EN) in these patients. The objective of this work was to assess the efficacy and tolerance of EN in children with BA awaiting LT.

**Methods:**

A total of 31 patients with BA followed between 1995 and 2018 were retrospectively included. Anthropometric indicators (weight, length, and head circumference) and adverse effects of EN were noted at the start (T0) and the end (T1) of EN. The *z*-scores for anthropometric indicators were compared between T0 and T1.

**Results:**

The median age at T0 was 7 months (interquartile range [IQR] 5–9), and the median duration of EN was 9 months (IQR 3–17). The *z*-scores for anthropometric variables improved from T0 to T1: –1.6 (IQR –2.5 to –1.0) to –0.5 (IQR –1.8 to 0.3) for median weight for age; –1.3 (IQR –2.4 to 0) to –0.4 (IQR –2.0 to 0.7) for length for age; –0.9 (IQR –2.3 to –0.3) to –0.3 (IQR –1.2 to 0.1) for weight for length; and –1.2 (IQR –2.1 to –0.6) to –0.2 (IQR –1.6 to 0.4) for body mass index (*p* < 0.05 for all comparisons). Nearly all (94%) of the patients had a weight-for-length *z*-score > –2 at the end of EN; 23% had adverse effects and 10% had complications leading to the cessation of EN.

**Conclusion:**

EN is effective and well tolerated in infants with BA awaiting LT.

## Introduction

Biliary atresia (BA) is the most common cause of neonatal cholestasis and the leading indication for liver transplantation (LT) in children (1, 2). The incidence of BA in France is 1 in 18,400 births, or about 41 children per year (2). BA involves the progressive fibrosis of the intra- and extrahepatic bile ducts and leads to obstruction (3, 4). Surgical treatment comprises the restoration of bile drainage using Kasai portoenterostomy (KPE).

A total of 70–80% of patients who undergo an operation for BA progress to biliary cirrhosis requiring LT (2, 5–7). Malnutrition is a common complication of cholestatic and end-stage liver diseases (8–11) and is related mainly to low-energy intake, altered nutrient metabolism (increased energy expenditure, insulin resistance, and abnormal oxidation of macronutrients), and malabsorption/maldigestion (1, 4, 12–14). Malnutrition is associated with increased morbidity and mortality following LT (1, 8, 12, 15–17) and with compromised neurocognitive development (12, 15, 18, 19). Nutritional management of children awaiting LT is therefore essential.

Due to the high energy needs in children with cholestasis, energy intake should be increased to 130–150% of the dietary reference values (DRVs) (1, 12). This objective is difficult to achieve through oral feeding alone (20). The use of enteral nutrition (EN) may help to ensure good nutritional status and therefore improve post-LT survival and neurocognitive development (12, 14, 21–23). Few studies have evaluated the efficacy of EN in patients with BA awaiting LT (12, 18, 20). The objective of this study was to assess the efficacy and tolerance of EN in children with BA awaiting LT.

## Materials and methods

### Study design

We conducted a retrospective, observational, and monocentric study. We analyzed data for patients who were operated on for BA in our tertiary hospital in Lille, France, between 1995 and 2018.

### Selection criteria

Every child operated on for BA between 1995 and 2018 in the Lille University Hospital who was awaiting LT and who received EN were included. The feed was started as soon as growth was faltering and was given overnight. Children who received parenteral nutrition (PN) as first-line treatment and children who were still receiving EN at the time of the study were not included.

### Study end points

The primary study end point was the success rate of EN defined *a priori* as a weight-for-length (W/L) or weight-for-height (W/H) *z*-score > –2 at the end of EN and a decrease of < 0.5 standard deviation (SD) between the start (T0) and end (T1) of EN. The secondary study end points were *z*-scores for the evolution of weight for age, height, or length for age, W/L or W/H, head circumference (HC), mid-upper arm circumference (MUAC), body mass index (BMI) at T0 and T1, and tolerance of EN.

### Data collection

Data were extracted from the national register for BA. The data were collected during medical consultations, assessments by dietitians, hospitalizations, and during home visits by the Home Artificial Nutrition Unit. The Home Artificial Nutrition Unit is a department of the Lille University Hospital that ensures the management of EN at home through a quarterly visit by a dietitian, who performs anthropometric measurements and evaluates energy intake and EN tolerance. The following information was collected:

– Date of birth, sex, weight (considered apart from ascites or edema conditions), length, and HC at birth, prematurity, and intrauterine growth retardation.
– Age at surgery and complications of surgery.
– T0 and T1 for EN, and reason(s) for stopping EN.
– Complications of EN, including vomiting, diarrhea, abdominal pain, inhalation, ascites, hemorrhage, or refeeding syndrome, and major complications of EN such as complications leading to the cessation of EN.

Clinical and biological parameters were collected at T0 and T1. Anthropometric parameters included weight, length, or height, W/L or W/H, BMI, HC, and MUAC. The following parameters were collected:

– Weight was measured without clothes, to the nearest 10 g.
– The length was measured in the supine position with a tape measure before 2 years of age.
– Height was measured while standing after age 2 years. HC was measured by encircling the supraorbital ridges and occipital protuberance.
– Microcrania was defined as an HC > 2 SD below the mean for sex and age.

Anthropometric parameters were expressed as *z*-scores according to the French reference growth charts of Sempé and Pédron (24). BMI was expressed as a *z*-score according to the French reference charts of Rolland-Cachera (25).

The following other information was also collected:

– Laboratory values such as conjugated bilirubin (μmol/L), prothrombin (%), albumin (g/L), and γ-glutamyl transferase (GGT) (U/L).
– Diuretic treatments.
– EN formula and daily caloric intake by EN and oral feeding as a percentage of the DRVs for age and sex (26).
– Presence of portal hypertension (splenomegaly, leukopenia, thrombocytopenia, and esophageal varices), edema of the lower limbs, or ascites (clinically and/or on ultrasound).

### Statistical analysis

Qualitative data are presented by frequency and percentage. Quantitative data are expressed as mean and SD and/or median and interquartile range (IQR). Anthropometric data were compared between T0 and T1 using the non-parametric matched Wilcoxon test. A *p*-value of < 0.05 was considered to be significant. Statistical analyses were performed using SAS software (version 9.4; SAS Institute, Cary, NC, United States) by the Biostatistics Unit of Lille University Hospital.

### Ethical aspects

In accordance with French laws, because this study was retrospective and observational, formal informed consent from the patients/parents and ethical committee approval were not required. All data were anonymized.

## Results

### Population

**FIGURE 1:**
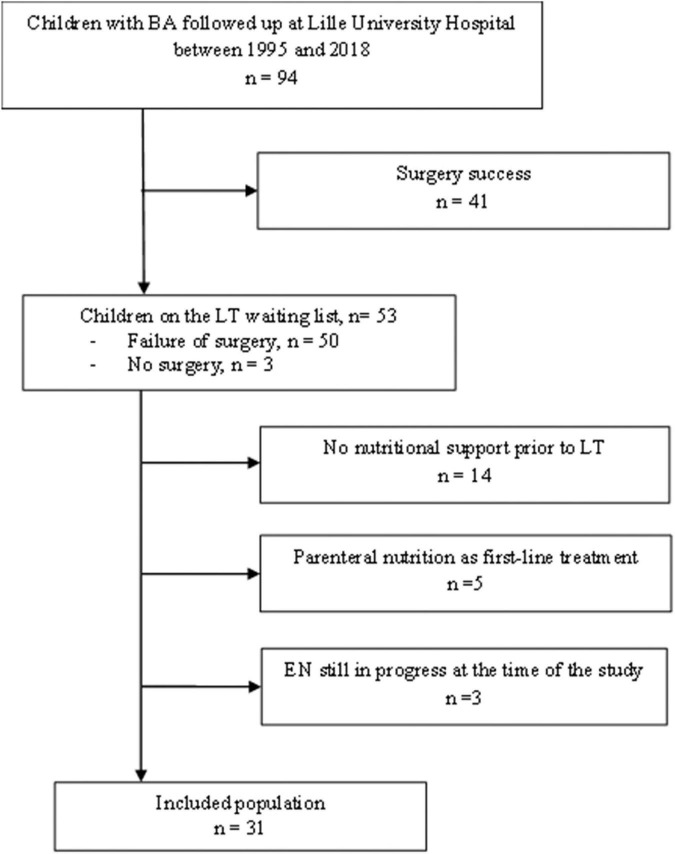
Study flow-chart. BA, biliary atresia; LT, liver transplantation.

A total of 94 patients with BA were followed up at the Lille University Hospital between 1995 and 2018. A total of 41 patients were successfully operated on and did not require LT (Figure 1). A total of 34 (36%) patients with BA received EN while awaiting LT: three were still receiving EN at the time of the study and were excluded from the analysis. Finally, 31 patients who received EN before LT were included. Most were girls (sex ratio 0.72). The number of patients included every year in the program (1–2 per year) did not change over the study period as well as the age when EN was started (7.5 months ± 2.3 SD vs. 6.2 months ± 3.1 SD, comparing the first 16 patients from 2008 to 2010, to the last 15 patients from the more recent period, *p* = 0.18). Only one patient had intrauterine growth retardation. The mean age at KPE was 56 days (± 19). Two patients were not operated on because of a delayed diagnosis.

### Patient characteristics at the start of enteral nutrition (T0)

The median age at T0 was 7 months (IQR 5–9). EN was started after the age of 1 year in only two patients. The median W/L or W/H *z*-score at T0 was –0.9 (IQR –2.3 to –0.3) and improved up to 0.3 at T1 (IQR –1.2 to 0.1). Notably, nine (29%) patients had a W/L or W/H *z*-score < –2, and ten (32%) patients had growth retardation (length- or height-for-age *z*-score < –2). All patients received semi-elemental formula except for one patient who received an amino acid-based formula because of an immunoglobulin E-mediated cow’s milk protein allergy that was resistant to hydrolyzed formula. The semi-elemental formula (Peptamen Junior, produced by Nestlé, Vevey, Switzerland) was isocaloric (1 kcal/ml) or hypercaloric (1.5 kcal/ml) with 60% of medium-chain triglycerides (MCTs) according to volume tolerance. The mean intake of protein/kg of body weight was 1.5 ± 0.7 SD. None of the patients had dextrin maltose or protein added to the formula. We chose to systematically start EN for a night administration for patient’s comfort and affect as less as possible their oral feeding intake during the day. When calculating the energy supply by EN, we anticipated a 10% reduction in the patient’s oral intake. Finally, we also adapted the caloric intake according to the evolution of their oral intake and their weight gain, which often ends in prolonging EN duration.

All patients had portal hypertension. A total of 14 (45%) patients received diuretic treatment (furosemide or spironolactone). The mean albumin concentration was 33 g/L (SD, 4), mean prothrombin time was 81% (SD ± 15), mean conjugated bilirubin was 234 μmol/L (SD, 104), and mean GGT concentration was 365 U/L (SD, 288).

### Success rate of enteral nutrition (W/H or W/L *z*-score > –2) at T1

Of 31 patients, 29 had a W/H or W/L *z*-score > –2 at T1, and EN failed in two patients, which meant that EN was successful in 94% of the patients. The first patient with EN failure had intrauterine growth retardation. He underwent a KPE at 48 days of age. EN was started 15 days after surgery because of poor oral intake but was not well tolerated, as shown by vomiting and bloating. Small-bowel X-ray showed a dilatation, which led to the suspicion of the subocclusive syndrome. However, the patient’s EN tolerance was not ameliorated after adhesiolysis, and EN was stopped at the age of 4 months and PN was started. The W/L *z*-score at T1 was –2.2. The second patient with EN failure had a syndromic BA involving interatrial communication, anomalous pulmonary venous return, and intestinal malrotation. He underwent heart surgery at the age of 4 months. EN was stopped at 6 months because of insufficient weight gain and PN was started. The patient’s W/L *z*-score at T1 was –2.1. This patient died at the age of 7.5 months from respiratory failure related to hepatopulmonary syndrome while awaiting LT.

### Evolution of anthropometric parameters between the start (T0) and end (T1) of enteral nutrition

**FIGURE 2:**
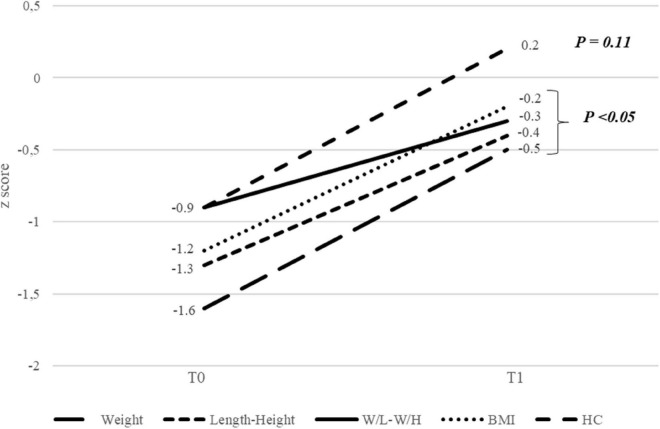
Evolution of anthropometric parameters between the start (T0) and end (T1) of enteral nutrition. W/L, weight for length; W/H, weight for height; BMI, body mass index; HC, head circumference.

**FIGURE 3:**
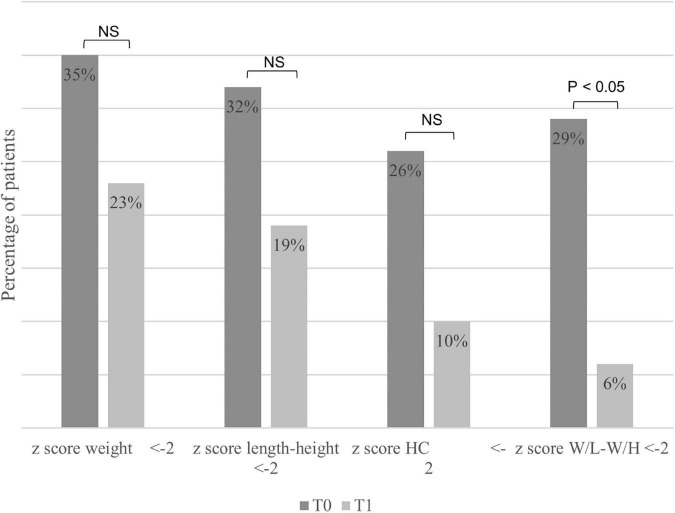
Percentage of patients with a z score < –2 for anthropometric parameters at the start (T0) and the end (T1) of enteral nutrition. HC, head circumference; W/L, weight for length; W/H, weight for height; NS, not significant.

The median duration of EN was 9 months (IQR 3–17), and the median age at T1 was 18 months (IQR 9–26). The *z*-score for weight, length, or height, W/L or W/H, and BMI improved significantly between T0 and T1 (*p* < 0.05) (Figure 2). The percentages of patients with a *z*-score < –2 decreased between T0 and T1 for weight, length, or height, HC, and W/L or W/H. The difference was significant for W/L or W/H (Figure 3). The evolution of MUAC between T0 and T1 could not be analyzed because of the extent of missing data at T1 (20/31 patients, 65% of patients with missing data).

### Evolution of caloric intake between T0 and T1

At T0, the mean oral caloric intake was 101% of the DRVs, and the mean total caloric intake (oral + enteral) was 186% of the DRVs. Between T0 and T1, oral intake decreased from 101% to 68% of the DRVs, and enteral intake increased from 85 to 91% of the DRVs. At T1, the mean total caloric intake was 159% of the DRVs.

### Complications and reasons for cessation of enteral nutrition

Of the patients, seven (23%) had complications; each patient could have several complications (Table 1). Of note, four (13%) patients had nausea or vomiting, two (6%) patients had diarrhea, and three (10%) patients frequently removed their nasogastric tubes. These complications occurred mainly at the start of EN and improved within a few weeks. In one patient with diarrhea and vomiting, the diagnosis of cow’s milk protein allergy was confirmed, and the symptoms regressed after the introduction of amino acid-based formula. In addition, three (10%) patients had major complications that led to the cessation of EN and the switch to PN, two (6%) patients had recurrent vomiting with bloating and discomfort, and one patient had fluid overload with respiratory distress; this patient had severe congenital anomalies associated with BA such as interatrial communication, ventricular septal defect, and bridging bronchus.

**TABLE 1:** Complications and reasons for cessation of enteral nutrition.

	Patients, *n* = 31 (%)
**Complications**:	**7 (23%)**
Nausea/vomiting	4 (13%)
Diarrhea	2 (6%)
Accidental removal of nasogastric tube	3 (10%)
Hemorrhage due to nasogastric tube	1 (3%)
Major complications:	**3 (10%)**
Recurrent vomiting, bloating, discomfort	2 (6%)
Respiratory distress, volume overload	1 (3%)
**Causes of cessation of enteral nutrition**:	
Liver transplantation	12 (39%)
Switch to parenteral nutrition	17 (55%)
Improvement of nutritional status	2 (6%)

No death caused by EN or refeeding syndrome was reported. EN was continued until LT in 12 (39%) patients. A switch to PN was necessary in 17 (55%) patients because of insufficient weight gain (*n* = 6), ascites (*n* = 5), hematemesis (*n* = 2), respiratory distress (*n* = 2), protein-losing enteropathy (*n* = 1), or recurrent cholangitis (*n* = 1). For two patients, EN was stopped because their nutritional status improved and they returned to full oral feeding until LT.

## Discussion

Our study found significant improvements in weight, length, or height, BMI, and W/L or W/H *z*-score between the start (T0) and end (T1) of EN in patients with BA awaiting LT. Most patients had a W/L or W/H *z*-score > –2 when EN was stopped. EN was generally well tolerated, and only 10% of patients had major complications that led to the cessation of EN.

Our study confirms the main findings of a previous study by Macías-Rosales et al. that compared the effectiveness of EN vs. oral nutrition for 12 weeks in 15 infants with BA (20). In the EN group, the *z*-scores for length and HC remained stable from T0 to T1: –2.4 to –2.2 (*p* = 0.697) and –1.9 to –2.1 (*p* = 0.650), respectively. These values decreased significantly in the oral nutrition group: –2.4 to –3.2 (*p* < 0.001) for length and –1.5 to –2.1 (*p* < 0.001) for HC (20). In a case series of five patients with chronic cholestasis who underwent percutaneous endoscopic gastrostomy, one boy with BA showed a weight gain of 3.1 kg after 10 months but also developed ascites and needed PN (27). Holt et al. showed that the linear growth and anthropometric measurements of 33 children with BA and failure to thrive improved after 3–7 months of nasogastric feeding (28). Yuksekkaya et al. found an improvement in MUAC after 2 months of EN in 38 children (median age 5.6 months ± 3.2) with neonatal cholestasis, including nine children with BA (15). Similarly, Charlton et al. reported significant improvements in the *z*-scores for weight and MUAC after 8 weeks of EN in 10 children with advanced cirrhosis, 6 of whom had BA (18).

Interestingly, 55% of our cohort finally required conversion to PN because of poor weight gain or ascites. This finding shows that PN may be an alternative in some patients and shows benefits by improving children’s nutritional status as reported by Wendel et al. (29). Sullivan et al. (30) also demonstrated better anthropometric outcomes when comparing 25 patients with BA who had PN and 22 patients without PN, with a mean duration of PN of 2.9 months, after a same mean duration of EN. Using PN is also recommended in case of EN failure by the 2019 guidelines published by Mouzaki et al. (12), as well as a recent review by Boster et al., who advised first using oral feeds, second nasogastric tube feeding when oral intake is insufficient, and third PN in case of poor growth despite NG feeds (31). Our results reinforce the importance to set up an early EN prior to transplantation in order to prevent the onset of malnutrition and consequences of advanced chronic liver diseases and avoid the use of PEN.

A new finding of our study is that, although not statistically significant, the rate of children with microcrania decreased from 26 to 10% from T0 to T1. This suggests a positive effect of improving nutrition on brain growth in these children. Infancy and early childhood (from birth to 24 months) are critical periods for neurodevelopment because of the rapid growth and differentiation of brain regions (32, 33). As the young brain accounts for 60% of the body’s energy consumption, malnutrition during early childhood may compromise neurodevelopment (32). Language and motor skills are impaired in children with BA awaiting LT, although the roles of chronic liver disease and prolonged hospitalization in these children are not clearly understood (34). Growth retardation before LT is an independent risk factor for long-term cognitive deficit (34–40).

In addition to being effective, EN was well tolerated. Most of the complications were transient and resolved quickly except in two patients. In the study by Macías-Rosales et al., diarrhea was experienced more often by patients in the EN group than in the oral nutrition group, but the rates of respiratory infections, vomiting, and abdominal distension did not differ between groups (20). Charlton et al. reported good tolerance to EN. During the 8 weeks of that study, all children tolerated a daily caloric intake of 140% of the DRVs (18).

As previously reported, our study found a high prevalence of malnutrition in children with BA despite optimized oral intake. All of our patients were under the care of a dietitian and received high-calorie formula (0.8–1.2 kcal/ml) before the start of EN. This prompted us to choose semi-elemental formulas enriched in MCT for most patients. In fact, in France, chronic liver disease-specific formulas enriched with branched-chain amino acids are not available. We were fully aware that children with an advanced BA should benefit from these formulas that were designed for cholestatic infants, as stated in the 2007 nutritional guidelines by Baker et al. (21). The only way to add MCT, which are necessary due to cholestasis and long-chain fatty acid malabsorption, was to use semi-elemental formulas that contain 60% of MCT. However, even with this fortified diet, the mean oral caloric intake was only 101% of the DRVs at T0. Macías-Rosales et al. found similar results (20). Shepherd et al. reported that oral caloric intake was 63% of the DRVs at the time of acceptance for LT (41). These findings indicate that appropriate caloric intake (130–150% of the DRVs) cannot always be achieved by optimizing the oral diet (1, 12, 21). EN made it possible to achieve the high caloric intake recommended in patients with BA (> 130% of the caloric requirements for age). Moreover, growth retardation occurs early. In this study, 32% of children had chronic malnutrition (length *z*-score < –2 SD) at a median age of 7 months.

The North American and European Societies for Pediatric, Gastroenterology, Hepatology and Nutrition recently proposed a practical approach to the nutritional support of children with the end-stage liver disease through the early onset of EN (12). EN on a nasogastric or nasojejunal tube is recommended in cases of poor tolerance or poor evolution of anthropometric parameters after 2–4 weeks of an optimized oral diet (e.g., fortified diet, use of medium-chain triglyceride-enriched formula, and smaller volumes more frequently) (12). These recommendations should lead to an earlier introduction of EN. Utterson et al. reported that only 16% of the 755 children awaiting LT had EN but that > 40% had growth failure (16). The early introduction of EN should help to improve the nutritional status before LT and, therefore, reduce the risk of morbidity and mortality following LT and improve brain development (8, 16, 17).

This study has both strengths and limitations. The strengths are the homogeneous population and follow-up of a large series of patients with BA. The limitations are the retrospective design and the missing data for MUAC at the follow-up. The study protocol, retrospective and over a long time period, does not allow to replicate our results. We, however, checked that there were no changes over time in the number of patients and the age of patients when starting EN. In fact, we included a mean of 1–2 patients per year over 23 years, and the age at T0 between the first 16 included patients (from 1999 to 2010) and the last 15 patients (from 2011 to 2018) was not statistically different (7.5 months ± 2.3 SD vs. 6.2 months ± 3.1 SD, *p* = 0.18). In patients with BA, weight measurement may be distorted by ascites, fluid overload, and/or organomegaly, which may lead to underestimation of malnutrition. We only recorded the presence/absence of ascites without grading its severity at T0 and T1. We, unfortunately, lack the INR values to calculate the PELD score, as well as MUAC values, for which we had 20 missing data, hindering us to use this parameter. In this context, MUAC is useful because it is less likely to be affected by fluid overload and is sensitive to short-term changes in nutritional status (12). We also did not collect EN modalities. In fact, recording the hours of EN administration is particularly relevant when the spontaneous oral intake tends to decrease over time in BA as in other chronic liver diseases.

## Conclusion

Enteral nutrition is effective and well tolerated in children with BA awaiting LT when commenced earlier. However, PN might still be required and then should be started without delay. EN significantly improves the *z*-score for weight, length, or height, W/L or W/H, and BMI. EN should be started early to prevent morbidity and mortality following LT and improve neurological development.

## Data availability statement

The raw data supporting the conclusions of this article will be made available by the authors, without undue reservation.

## Ethics statement

Ethical review and approval was not required for the study on human participants in accordance with the local legislation and institutional requirements. Written informed consent from the participants or their legal guardian/next of kin was not required to participate in this study in accordance with the national legislation and the institutional requirements.

## Author contributions

EP contributed substantially to the design of the work, the acquisition, analysis, and interpretation of data, wrote the draft, approved the final version of the manuscript, and agreed to be responsible for all aspects of the work by ensuring that the issues related to the accuracy or integrity of any part of the work are appropriately investigated and resolved. MA, DL, LT, SC, DG, DT, and FG contributed substantially to the design of the work, the analysis and interpretation of data, revised the manuscript critically, approved the final version of the manuscript, and agreed to be responsible for all aspects of the work by ensuring that the issues related to the accuracy or integrity of any part of the work are appropriately investigated and resolved. All authors contributed to the article and approved the submitted version.
